# Validation of the brief international cognitive assessment for multiple sclerosis (BICAMS) in the Portuguese population with multiple sclerosis

**DOI:** 10.1186/s12883-018-1175-4

**Published:** 2018-10-17

**Authors:** Cláudia Sousa, Mariana Rigueiro-Neves, Telma Miranda, Paulo Alegria, José Vale, Ana Margarida Passos, Dawn Langdon, Maria José Sá

**Affiliations:** 1 MS Clinic, Department of Neurology, Centro Hospitalar São João Porto, Alameda Prof. Hernâni Monteiro, 4200 – 319 Porto, Portugal; 2 BRU-IUL, Instituto Universitário de Lisboa (ISCTE-IUL), Lisbon, Portugal; 3 Department of Neurology, Hospital Beatriz Ângelo, Loures, Portugal; 4 Department of Psychology, Royal Holloway, University of London, London, UK; 5 Faculty of Health Sciences, University Fernando Pessoa, Porto, Portugal

**Keywords:** Multiple sclerosis, Cognitive impairment, BICAMS, Normative values for Portugal

## Abstract

**Background:**

The validation of international cognitive batteries in different multiple sclerosis (MS) populations is essential. Our objective was to obtain normative data for the Portuguese population of the Brief International Cognitive Assessment for Multiple Sclerosis (BICAMS) and assess its reliability.

**Methods:**

The BICAMS was applied to 105 MS patients and 60 age, gender and education matched healthy controls (HC). In order to test its reliability, BICAMS was re-administered in a subset of 25 patients after a 7-month interval.

**Results:**

Most participants were women, with a mean age of 37, 21 years and a mean of 14,08 years of education. The vast majority of the MS patients (92.4%) had the relapsing remitting type, 58.1% were professionally active, mean disease duration was 6.52 years, median EDSS score was 1.5 (range: 0–6.0) and the median MSSS score was 2.01 (IQR range: 3.83). The MS group presented significantly higher scores of anxiety and depression than HC and 47,4% had fatigue. The MS group performed significantly worse than the control group across the three neuropsychological tests, yielding the following values: SDMT: *t*(165) = 3.77, *p* = .000; CVLT-II: *t*(165) = 2.98, *p* = .003; and BVMT-R: *t*(165) = 2.94, *p* = .004. The mean raw scores for Portuguese normative data were as follows: SDMT: 58.68 ± 10.02; CVLT-II: 60.47 ± 10.12; and BVMT-R: 24.68 ± 5.52. Finally, test–retest reliability coefficients for each test were as follows: SDMT: *r* = .90; CVLT-II: *r* = .71; and BVMT-R: *r* = .84.

**Conclusions:**

The Portuguese version of BICAMS here in described is a reliable monitoring instrument for identifying MS patients with cognitive impairment.

## Background

Multiple sclerosis (MS) is a chronic inflammatory demyelinating disease of the central nervous system that can impair any body function, including cognition [1]. Cognitive dysfunction affects 40 to 70% patients [2, 3]. Irrespective of age and gender [3, 4], may occur at all stages of the disease, even at the very early beginning [5, 6] and definitely impacts the lives of MS patients and their families [3, 7, 8].

The characteristic pattern of cognitive impairment in MS has been described early on to include memory, information processing efficiency, executive functioning, attention and processing speed [1]. However, the cognitive domains most likely to be affected in MS are information processing speed and memory, whilst visual processing and executive function are less likely to be impaired and language is largely intact [1, 9–12].

The most frequently used neuropsychological batteries for patients with MS such us, the Brief Repeatable Battery of Neuropsychological tests and the Minimal Assessment of Cognitive Function in MS, require specialized technical and human resources and take a considerable time for evaluation in the daily clinical setting [1, 13]. Recently, the Brief International Cognitive Assessment for MS (BICAMS) was developed and recommended as a validated and standardized international screening test, because it is an easier assessment tool that can be administered by a technician who is not a specialist in neuropsychology and lasts only about 15 min to apply [14, 15]. Besides, the three instruments that compose BICAMS – Symbol Digit Modalities Test (SDMT) [16], California Verbal Learning Test (CVLT-II) [17] and Brief Visuo-spatial Memory Test Revised (BVMT-R) [18] – have previously been shown to have good psychometric properties.

The aims of this study are to describe the normative values of the Portuguese version of the BICAMS with gender, age and education corrections and to test the validity of this battery in a sample of Portuguese patients with MS.

## Methods

### Participants

A group of 105 patients with MS diagnosed according to the McDonald criteria [19] and a control group of 60 age, gender and education matched healthy subjects (HC), entered this study, and conducted in the period 2015–2016.

The MS patients were consecutively recruited at the MS Clinics from two hospitals located in separate regions of the country, Hospital de São João (Oporto; North) and Hospital Beatriz Ângelo (Loures; South), whereas the HC group was recruited from the community and among relatives and friends of MS patients. All participants were aged between 17 and 69 years and they were fluent in Portuguese as first language.

Exclusion criteria were current or past neurological disorder other than MS, presence of major psychiatric illness, history of learning disability, history of serious head trauma, presence of alcohol or drug abuse, relapse and/or corticosteroid use within 4 weeks preceding the neuropsychological assessment. HC were also required to present scores > 21 on Montreal Cognitive Assessment Portuguese version (MoCA) [20, 21].

The study was approved by the ethical committees of both hospitals. All the participants, from MS group and HC, volunteered to participate in this study, giving written informed consent.

### Procedures

An initial demographic interview was conducted. This was based on a common script that included a demographic questionnaire, medical history, drinking and drug habits and present health status. The MS data, such as type, duration, and degree of disability and severity, as assessed by the Expanded Disability Status Scale (EDSS) [22] score and the Multiple Severity Status Score (MSSS) [23], respectively, were obtained in the clinical protocols.

Then, participants underwent the BICAMS battery [14], which included the oral version of Symbol Digit Modalities Test (SDMT) [16], the learning trials from the California Verbal Learning Test-II (CVLT-II) [17] and the Brief Visuo-spatial Memory Test-Revised learning trials (BVMT-R) [18].

The SDMT [16] examines sustained attention, concentration and processing speed. In the oral version, the participant examines a series of nine meaningless geometric symbols, which are labeled from 1 to 9. Then, during 90 s the participant is instructed to say the corresponding number to each symbol, as rapidly as possible. The test score corresponds to the number of correct responses.

The CVLT-II [17] is a measure of verbal learning and memory. The test begins with the examiner reading a list of 16 words to the patient and then he/she is asked to report as many of the items as possible, in any order. After recall is recorded, the entire list is read again followed by a second attempt at recall. Altogether, there are five learning trials. The outcome measure is the total number of recalled items over the five learning trials.

The BVMT-R [18] is a measure of visuo-spatial learning and memory. The participant is exposed to a matrix of six simple abstract designs for 10 s followed by an unaided recall; we used the form 1 of the original test. After that, the participant is asked to render the designs using paper and pencil, taking as much time as needed for reproduction. The scoring criterion is based on location and accuracy of each design (from 0 to 2, maximum total score for each array 12). The outcome measure of this test corresponds to the total recall score across the three trials.

The validation was conducted per the international standards given by the expert consensus committee [15]. As the first step, the CVLT-II list of words were translated and re-translated from English to Portuguese and vice versa respectively; the other two tests did not require translation due to their nature. In the second step, the test instructions were translated into Portuguese.

In both groups, anxiety and depression symptoms were also measured using the Portuguese version of Hospital Anxiety and Depression Scale (HADS) [24]. In the MS group the level of fatigue was measured with the Modified Fatigue Impact Scale (MFIS) [25–27].

“The participants of both groups were asked to return for a follow-up session to allow for test–retest reliability analyses. A subgroup of 26 patients and 13 HC returned after a mean time of 7 months and all the tests administered in the first session were repeated in the same manner and in the same order.”

Well-trained clinical psychologists conducted all sessions and the tests were applied in a standardized way and in a fixed order. The mean time for BICAMS application was 15 min, as described [14, 15].

### Statistical analysis

Statistical analysis was performed using the Statistical Package for the Social Sciences (IBM SPSS), version 23.0. Descriptive statistics (e.g., mean, standard deviation, median, interquartile range and percentages) were used for demographic characterization of both groups. Student’s t-test for independent samples was used to analyze the differences between groups, at the level of *p* < .05. The values shown in the tables are bilateral *p*-values. The effect sizes of those differences were calculated using Cohen’s *d*. Spearman’s correlations (Ρ) were used to analyze reliability measures and the relationship between BICAMS, HADS and MFIS results. Raw scores were analyzed for the full sample and Z-scores were calculated. Multiple regression analysis was used to produce normative data.

## Results

### Demographics and MS characteristics

The groups were similar with regard to age (MS group: M = 38.26 years±11.03; HC: M = 36.17 years ±12.01, *p* = .63), gender (MS group: %Female = 66.7; HC: %Female = 58.3, *p* = .28) or number of educational years (MS group: M = 13.55 ± 3.71; HC: M = 14.62 ± 3.47, *p* = .42). With respect to professional status, the majority of subjects were employed, with a much higher proportion of HC than MS, as is usually reported (*n* = 56, 94.9%; *n* = 61, 58.1%, respectively). In the MS group, 92,4% (*n* = 97) of patients had the relapsing remitting type and 3,8% (*n* = 4) secondary progressive type and 3,8% (n = 4) clinically isolated syndrome. The average disease duration was 6.52 years (SD = 5.95) and the median EDSS score was 1.5 (range: 0–6.0). The MSSS score, calculated in patients from 1 to 30 years of disease duration (*n* = 95), had a median value of 2.01 (IQR range: 3.83).

### Criterion-related validity: Group differences

Means, standard deviations and *t test’s* for independent samples from the three tests are presented in Table 1. The results showed that MS group performed significantly worse than the HC group on all measures. Cohen’s *d* was analyzed for each neuropsychological test and were satisfactory: SDMT - 0.65 (large); CVLT-II - 0.49 (medium); BVMT-R - 0.45 (medium) [28].

**Table 1:** Group differences on BICAMS measures

	MS (*N* = 105)	HC (*N* = 60)	*t*	*P*
SDMT	51.77 (11.20)	58.68 (10.02)	3.77	0.000
CVLT-II	55.05 (11.84)	60.47 (10.12)	2.98	0.003
BVMT-R	21.72 (7.27)	24.68 (5.52)	2.94	0.004

### Reliability: Test-retest

The test–retest reliability data obtained in a subgroup of MS patients are presented in Table 2. The test-retest reliability coefficients showed a strong to a very strong and significant effect for all BICAMS tests.

**Table 2:** Test–retest means and correlations for MS group (*n* = 26)

	*Time 1*		*Time 2*		*Spearman’s correlation*	*P*
	*Mean*	*SD*	*Mean*	*SD*		
SDMT	*50.96*	*11.56*	*53.92*	*13.99*	*0.90*	*< 0.001*
CVLT-II	*57.08*	*12.75*	*57.31*	*17.44*	*0.71*	*< 0.001*
BVMT-R	*22.00*	*7.43*	*25.12*	*6.94*	*0.84*	*< 0.001*

The test-retest results in the HC were not considered in view of the low number of cases.

### Regression based-norms

To obtain a regression-based normative model for BICAMS, the distribution of the SDMT, CVLT-II and BVMT-R raw scores was analyzed for the complete sample and the Z scores were calculated. The raw scores were then converted into scaled scores (M = 10 and SD = 3), as presented in Table 3. For each test a multiple regression analysis with a stepwise method using the scaled scores as dependent variable and age, gender and education as predictors was performed. Education was introduced as the number of regular academic school years that the participant successfully completed. As some studies suggest that there is a curvilinear relationship between demographic variables and cognitive function [29], the quadratic term of age and education were also introduced as predictors. These results allow us to detect which variables contributed significantly to explain each of the scaled neuropsychological test scores.

**Table 3:** Raw score to scaled score conversions for the BICAMSs tests

Scaled score	SDMT	CVLT-II	BVMT-R
1	–	0–21	–
2	0–20	22–27	1–3
3	21–24	28–33	4–5
4	25–27	34–37	6–8
5	28–33	38–41	9–10
6	34–37	42–44	11–14
7	38–40	45	15
8	41–45	46–49	16–17
9	46–49	50–53	18–20
10	50–53	54–57	21–22
11	54–57	58–60	23–25
12	58–61	61–64	26–27
13	62–65	65–68	28–30
14	66–69	69–72	31–32
15	70–73	73–76	33–34
16	74–76	77–79	–
17	77–78	–	–

The T-scores corrected for education, age and gender were generated through a procedure suggested by Diehr and colleagues [30]. Therefore, another multiple regression (enter method) with each of the BICAMS test scaled scores as the dependent variable and the significant predictors of each test was performed. The non-standardized predicted values of this equation were saved and a new variable was calculated corresponding to the difference between an individual’s actual and predicted scale score (i.e., the residual) divided by the standard deviation of those residuals. These values were then rescaled for a T-score (M = 50 and SD = 10).

Finally, another multiple regression analysis with corrected T-score as the dependent variable was performed to generate each test normative formula for the Portuguese population. The final formula to calculate the T-scores for each of BICAMS’s test are presented below:

$$ \mathbf{SDMT}\ \mathrm{T}\ \mathbf{score}=10.511+\left({0.007}^{\ast }\ {\mathrm{age}}^2\right)+\left(-{0.966}^{\ast }\ \mathrm{years}\ \mathrm{of}\ \mathrm{education}\right)+\left({4.138}^{\ast }\ \mathrm{scaled}\ \mathrm{score}\right) $$

$$ \mathbf{CVLT}-\mathbf{II}\ \mathrm{T}\ \mathbf{score}=3.195+\left({0.006}^{\ast }\ {\mathrm{age}}^2\right)+\left({3.761}^{\ast }\ \mathrm{scaled}\ \mathrm{score}\right) $$

$$ \mathbf{BVMT}-\mathbf{R}\ \mathrm{T}\ \mathbf{score}=-8.004+\left({0.514}^{\ast }\ \mathrm{age}\right)+\left({\mathrm{3,833}}^{\ast }\ \mathrm{scaled}\ \mathrm{score}\right) $$

In determining impairment, the 5th percentile value based on the performance of healthy control sample was calculated for each test. Participants were considered impaired if their score was equal of below the percentile 5^th^of the control group (results are presented on the Table 4) [31]. Then, using the previously reported criteria of impairment defined by “one or more abnormal tests” [32, 33], it was found that 24.8% of the MS sample was impaired at baseline.

**Table 4:** The prevalence of cognitive impairment in MS patients according to the 5th percentile value of HC on BICAMS tests

	5th Percentile value for HC on each test	Percentage of MS patients under 5th percentile
SDMT	38	14.3%
CVLT-II	41	9.5%
BVMT-R	12	11.4%

Analysing the degree of disability assessed by EDSS and cognitive performance, we found significant correlations with all cognitive tests (SDMT: −.497, *p* = .000; CVLT: −.334, p = .000; BVMT: −.275, *p* = .005).

Regarding anxiety and depression symptoms, it was found that MS group presented higher scores on these measures than HC, and that these differences were statistically significant: anxiety (MS group: M = 7.85 ± 4.51; HC: M = 6.32 ± 3.00, *t* = − 2.348; *p* = .20) and depression (MS group: M = 5.14 ± 3.95; HC: M = 3.18 ± 2.57). Anxiety symptoms were found to be more frequent (*n* = 56; 53.3%) than depression symptoms (*n* = 29; 27.6%) in MS patients. In the MS group depression symptoms had a modest significantly negative effect only on CVLT-II results (R – 0.196; *p* = .45), whereas anxiety was not significantly correlated with any BICAMS test. The assessment with the MFIS scale (*n* = 95) showed that fatigue was present in 50 MS patients (47,4%) and was significantly correlated with the EDSS score (R – .279; *p* = .006), and with anxiety (R – .631; *p* = .0001) and depression symptoms (R – .754; *p* = .0001). Conversely, fatigue was negatively correlated with SDMT score (R – .266; *p* = .009); similar results were observed in both MFIS subscales, physical (M = 18.04 ± 9.66; R-.289; *p* = .005) and cognitive (M = 17.84 ± 10.21; R-.203; *p* = .049).

## Discussion

An international consensus committee of experts recently recommended a short battery of tests for cognitive assessment in MS that allows monitoring of cognition over time and is a fast and reliable instrument that may be administered by healthcare professionals with no specific experience in neuropsychological testing. According to the international standards for validation [15], several validation studies of BICAMS have been carried out in different cultures and languages, with the aim of making this psychometric tool more solid and internationally applicable. Up to now, there exists normative data for populations of several countries, such as Czech Republic [32], Italy [34], Hungary [35], Ireland [36], Brazil [37], Lithuania [38] Argentina [39], Canada [33], Greece [31], Belgium [40], Japan [41] and Turkish [42].

The current study followed the recommendations and standards of the BICAMS consensus committee [14, 15] and is the first to publish the Portuguese normative data for SDMT, CVLT-II and BVMT-R. Our results showed that MS group performed significantly worse than HC group on all measures (SDMT, CVLT-II and BVMT-R), a finding that is in agreement with the other recently published validations. These differences were more marked in the SDMT and CVLT-II than the BVMT-R, and similar results were found by O’Connell and colleagues (2015), Spedo and colleagues (2015) and Vanotti and colleagues (2016). Test-retest reliability in our population fits the recommended international standards for BICAMS validation [14]. Test–retest reliability for raw scores was adequate to excellent for all the three tests in this validation; more than .80 in SDMT and BVMT, replicating prior finds [33, 34]. Yet our results are lower than those of Vanotti and colleagues (2016). In addition we confirmed that the SDMT has particularly high test-retest reliability. We used a wider time span than other authors [37, 40] in order to avoid the learning effect, since at both evaluation times the same forms were applied.

The BICAMS tasks were able to identify cognitive impairment in 24.8% of MS patients using the criteria of impairment defined by one or more abnormal tests. This is a lower value than those found in other studies, which ranged from 47.3 to 58% [31–33, 35, 36]. This result may reflect the characteristics of our MS sample, which were mainly RRMS and rather early cases (mean disease duration 6.5 years) and a correspondingly low level of physical disability, median EDSS 1.5 [31–33, 36]. The lower level of disability in our sample is further supported by our MSSS data [23].

We found a significant correlation between EDSS and cognitive performance in the three tests used, that is, the higher the EDSS score the worse the cognitive test performance.

Regarding anxiety and depression symptoms, we found that the MS group also presented with higher scores on these measures then the HC, fitting the results of other BICAMS validation studies [32, 33, 37]. The Hungarian BICAMS validation reported a negative correlation of fatigue with all BICAMS tests [31]. In our study an association with fatigue was only seen in the SDMT test, possibly reflecting the lower fatigue in our patients as well as the lower physical disability.

This study was some limitations. First, follow-up assessments were done in a low number of cases, especially in the HC group, which is due to the fact that some individuals live far from the Hospital and incur additional personal costs. Another limitation is the fact that effect size for CVLT and BVMT-R although satisfactory, is on the threshold of the effect size classified as medium.

## Conclusions

In conclusion, our study provides the Portuguese BICAMS standards for use with MS patients and evidences the strong psychometric properties of the Portuguese BICAMS version. The normative data of the BICAMS for the Portuguese population enables the use of the battery in clinical practice, for longitudinal patient assessments and as an outcome measure of cognitive functioning in clinical trials. Future prospective studies with larger samples of MS patients, with different types of disease evolution, will certainly add valuable information concerning the clinical applicability of the Portuguese BICAMS version.

## Abbreviations

BICAMS: Brief International cognitive assessment for multiple sclerosis
BRB-N: Brief repeatable battery of neuropsychological tests
BVMT-R: Brief visuo-spatial memory test – revised
CI: Cognitive impairment
CVLT-II: California verbal learning test – II
EDSS: Expanded disability status scale
HADS: Hospital anxiety and depression scale
HC: Healthy subjects
IBM SPSS: Statistical package for the social sciences
MACFIMS: Minimal assessment of cognitive function in multiple sclerosis
MFIS: Modified fatigue impact scale
MoCa: Montreal cognitive assessment
MS: Multiple sclerosis
MSSS: Multiple severity status score
SDMT: Symbol digit modalities test
